# ATR suppresses the pro-tumorigenic functions of breast stromal fibroblasts

**DOI:** 10.18632/oncotarget.26159

**Published:** 2018-10-05

**Authors:** Mysoon M. Al-Ansari, Abdelilah Aboussekhra

**Affiliations:** ^1^ Department of Molecular Oncology, King Faisal Specialist Hospital and Research Center, Riyadh, Saudi Arabia; ^2^ Department of Microbiology, Faculty of Science and Medical Studies, King Saud University, Riyadh, Saudi Arabia

**Keywords:** ATR, breast cancer, cancer-associated fibroblasts, p53

## Abstract

The ATR protein kinase is a master regulator of the cellular responses to DNA damage and replication stresses. Despite these crucial physiological roles, the implication of ATR in human carcinogenesis remains elusive. We have shown here that the ATR level is reduced in most cancer-associated fibroblasts (CAFs) as compared to their adjacent normal counterparts. Importantly, specific *ATR* knockdown activated breast fibroblasts, and enhanced their paracrine pro-carcinogenic effects via strong increase in the expression/secretion of SDF-1 and IL-6. Furthermore, ATR-deficient fibroblasts enhanced tumor growth and aggressiveness in orthotopic breast tumor xenografts. On the other hand, ectopic expression of *ATR* suppressed the expression/secretion of several cancer-promoting proteins such as IL-6, TGF-β1 and SDF-1, and inhibited the migration and invasion capacities of breast myofibroblast cells. Furthermore, *ATR* up-regulation in active breast fibroblasts reduced their paracrine pro-migratory/-invasive effects on breast cancer cells. Interestingly, the cancer promoting effects of ATR-deficient cells were repressed by ectopic expression of the ATR effector p53. These results indicate that ATR is a major target of cancer cells in breast fibroblasts wherein this protein kinase represses both autocrine and paracrine pro-carcinogenic effects. This indicates that the ATR status in these cells could be of great prognostic/diagnostic values.

## INTRODUCTION

Several lines of evidence indicate that cancer-associated fibroblasts (CAFs), which constitute the major component of the tumor stroma, actively participate in breast tumor growth and spread [1, 2]. Active fibroblasts or myofibroblasts promote tumor growth through secreting several soluble pro-carcinogenic factors such as SDF-1 and IL-6 [3, 4]. The expression of these factors is repressed by several tumor suppressor proteins, such as p16 and p53, which are cell cycle checkpoint proteins with non-cell-autonomous tumor suppressive function as well [5, 6]. Activation of the p53 pathway occurs in response to cellular stresses, which appear to be dependent on distinct upstream regulatory kinases. One of these important protein kinases is the Ataxia-Telangiectasia and Rad-3 related (ATR) protein. The ATR protein kinase is a cell cycle checkpoint protein, which coordinates cellular responses to DNA damage and replication stress, and therefore it is essential for the maintenance of genomic integrity [7]. The *ATR* gene is essential, and its disruption leads to early embryonic lethality in mice and formation of large benign tumors in heterozygote animals [8, 9]. However, hypomorphic mutations in the gene, with only partial loss of function, lead to the rare hereditary disorder Seckel syndrome [10, 11]. Based on several studies, ATR is not mutated in the vast majority of human cancers, including breast and ovarian carcinomas [12]. However, changes in the expression level of this protein kinase could play a major role in carcinogenesis.

In this study, we addressed the role of ATR in regulating the activity of breast stromal fibroblasts and their paracrine procarcinogenic effects. We have clearly shown that ATR has non-cell-autonomous tumor suppressive functions both *in vitro* and in animal models.

## RESULTS

### ATR is down-regulated in active breast cancer-associated fibroblasts

**Figure 1:**
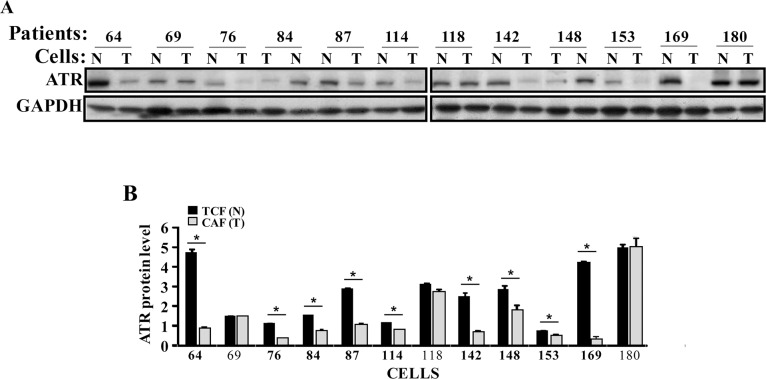
ATR is down-regulated in CAFs **(A)** Whole cell lysates were prepared from the indicated CAF (T) and TCF (N) cells and 50 μg of proteins were used for immunoblotting analysis using antibodies against the indicated proteins. **(B)** Histogram, the values were determined by densitometry following immunoblotting and normalized against GAPDH. Error bars indicate mean ± SD for 3 different experiments, ^*^P < 0.001.

We started the present study by assessing the ATR expression level in 12 human breast CAFs and their counterpart fibroblasts isolated from histologically normal adjacent cancer-free tissues (TCFs). CAF/TCF pairs were always used simultaneously at similar passages. Whole cell extracts were prepared and specific anti-ATR and anti-GAPDH (used as internal control) antibodies were utilized for immunoblotting analysis. Figure 1A and 1B shows that the ATR level is lower in 9 out of 12 CAFs (75%) as compared to their corresponding TCFs. However, ATR level was similar in 3 CAF/TCF pairs, namely 69, 118 and 180 (Figure 1A-1B). Furthermore, a great inter-individual variation in ATR expression was observed between the various CAFs and also TCFs (Figure 1A-1B).

### ATR down-regulation activates breast stromal fibroblasts

**Figure 2:**
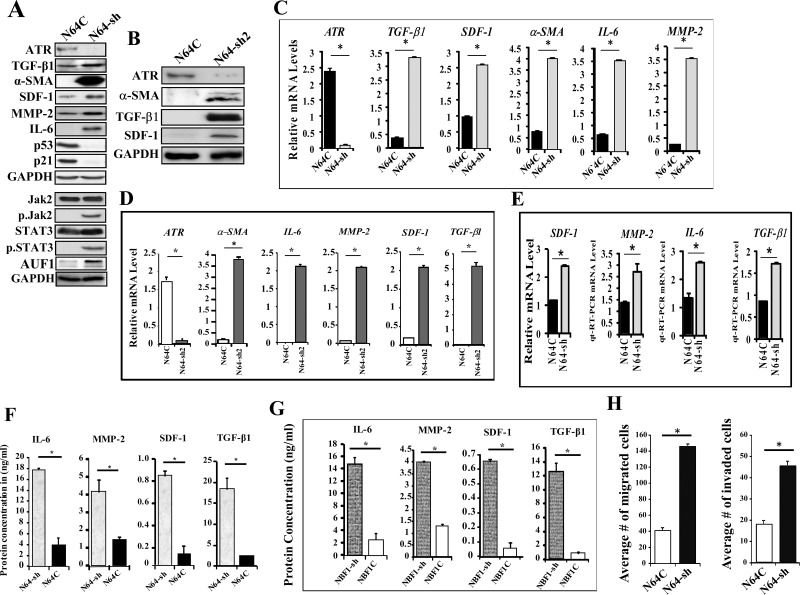
ATR down-regulation activates breast stromal fibroblasts TCF-64 cells were transfected with specific ATR-shRNA (N64-sh) or a scrambled sequence (N64C). **(A** and **B)** Whole cell lysates were prepared from the indicated cells and were used for immunoblotting analysis using antibodies against the indicated proteins. **(C)** Total RNA was extracted from the indicated cells, and the mRNA levels of the indicated genes were assessed by qRT–PCR using specific primers. Error bars represent mean ± S.D. ^*^P < 4x10^−6^. **(D)** Total RNA was extracted from the indicated cells, and the mRNA levels of the indicated genes were assessed by qRT–PCR using specific primers. Error bars represent mean ± S.D. ^*^P < 4x10^−6^. **(E)** N64-sh and N64C cells were seeded separately over 3D networks Biotech in 24 well plate, and then were allowed to grow under observation. After 10 days, total RNA was extracted, and the mRNA levels of the indicated genes were assessed by qRT-PCR. Error bars represent mean ± S.D. ^*^P< 0.031. **(F** and **G)** SFCM were collected from the indicated cells and the levels of the indicated proteins were determined by ELISA. Error bars represent mean ± S.D (n=3). ^*^P < 0.017811 and ^*^P< 0.003, respectively. **(H)** Cells (10^5^) were seeded in SFM onto the upper compartment of the migration and invasion plates and incubated for 24 h in the presence of CpM in the lower chambers of 24-well BD BioCoat plates. The numbers of migrated/invaded cells were calculated and represented in histograms. Error bars represent mean ± SD (n=3). ^*^P < 3.1x10^−5^.

To evaluate the autocrine and the paracrine effects of ATR down-regulation in breast stromal fibroblasts, we knocked-down ATR using specific shRNA in TCF-64 cells, while a scrambled sequence was used as control. Figure 2A shows that the ATR protein level was reduced in the *ATR*-shRNA-treated cells (N64-sh) as compared to their corresponding control cells (N64C). This decrease was accompanied with a strong increase in the levels of α-SMA, SDF-1, IL-6 and TGF-β1 (four important markers of active stromal fibroblasts) (Figure 2A). Similar results were obtained using another *ATR*-shRNA sequence (N64-sh2) (Figure 2B). Likewise, ATR down-regulation increased the expression of MMP-2 (Figure 2A). However, ATR down-regulation reduced the level of the tumor suppressor protein p53 and its target p21 (Figure 2A). Moreover, ATR deficiency activated Jak2 and STAT3, which led to the up-regulation of their downstream target AUF1 (Figure 2A). These results suggest that ATR represses breast stromal fibroblasts through inhibition of the Jak2/STAT3 pathway.

Figure 2C shows that the mRNA levels of the *TGF-β1*, *SDF-1*, *α-SMA*, *IL-6* and *MMP-2* genes were strongly increased in ATR-defective cells as compared to their corresponding control cells. Similar results were obtained using ATR-shRNA2 in TCF-64 (Figure 2D). These genes were also up-regulated in ATR-deficient cells (N64-sh) as compared to controls (N64C), when these cells were grown in 3D culture conditions (Figure 2E).

We have next assessed the effect of ATR down-regulation on the secretion of some important cytokines. Therefore, N64-sh and N64C cells were cultured in SFM for 24 h, and the resulting SFCM were collected and the levels of the secreted proteins were assessed by ELISA. Figure 2F shows that the secreted levels of the MMP-2, SDF-1, IL-6 and TGF-β1 proteins significantly increased in N64-sh cells as compared to N64C. Similar results were obtained when ATR was knocked-down using ATR-shRNA1 in normal breast fibroblast cells (NBF-1) (Figure 2G). This indicates that ATR negatively controls the expression/secretion of these cancer-promoting proteins in breast stromal fibroblasts.

We have next investigated the effect of ATR down-regulation on their invasion and migration abilities. Figure 2H shows that the number of migrating and invading ATR-deficient cells (N64-sh) was significantly higher than the number of migrating and invading control cells (N64C). This indicates that ATR represses both the migratory and the invasiveness abilities of breast stromal fibroblasts.

### ATR inhibits the paracrine pro-carcinogenic effects of breast stromal fibroblasts through suppressing the expression/secretion of SDF-1 and IL-6

**Figure 3:**
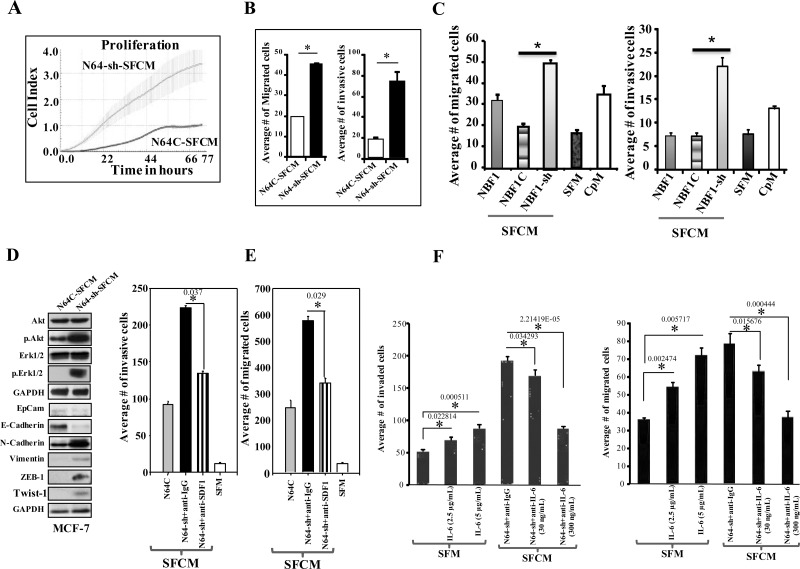
ATR down-regulation enhances the paracrine pro-carcinogenic effects of breast stromal fibroblasts in an SDF-1/IL-6-dependent manner **(A)** SFCM collected from the indicated cells were added separately to MCF-7 cells previously seeded into 96 wells, and cell proliferation was assessed by the real-time cell electronic sensing system. **(B)** MCF-7 cells were seeded onto the upper compartment of the migration and invasion plates, and then were incubated for 24 h in the presence of SFCM from the indicated cells. Error bars represent mean ± S.D (n=3). ^*^P< 0.0002. **(C)** MDA-MB-231 cells were seeded onto the upper compartment of the migration and invasion plates, and then were incubated for 24 h in the presence of SFCM from the indicated cells. SFM and CpM were used as controls. Error bars represent mean ± S.D (n=3). ^*^P< 1.7x10^−3^. **(D)** Whole cell lysates were prepared from MCF-7 cells that were treated for 24 h with SFCM collected from the indicated cells. Immunoblotting analysis was performed using the indicated antibodies. **(E** and **F)** MCF-7 cells were seeded onto the upper compartment of the migration and invasion plates, and then were incubated for 24 h in the presence of the indicated media. Neutralizing antibodies (anti-SDF-1, anti-IL-6) or recombinant human IL-6 protein were separately added to SFCM or SFM, respectively. IgG was utilized as control. The numbers of migrated and invaded cells were represented in histograms. Error bars represent means ± S.D (n=3). ^*^P-values are indicated.

In addition, we have utilized SFCM from N64-sh cells (N64-sh-SFCM) and N64C cells (N64C-SFCM) to treat MCF-7 cells in 96-well plates. The real-time cell electronic sensing system was used to monitor the effect of each SFCM on cellular proliferation. Figure 3A shows that N64-sh-SFCM enhanced the proliferation rate of MCF-7 cells more than 4 fold as compared to cells grown in the presence of N64C-SFCM. Similarly, the migration and invasion abilities of MCF-7 cells were 2.25 and 3.75 fold higher in the presence of N64-sh-SFCM than in the presence of N64C-SFCM, respectively (Figure 3B). Similar results were obtained with MDA-MB-231 cultured with SFCM derived from NBF1-sh1 or NBF1C (Figure 3C). This indicates that ATR-deficient breast stromal fibroblasts enhance the proliferation, migration and invasion abilities of breast cancer cells through paracrine secreted factors.

Subsequently, the level of the active forms of Erk1/2 and Akt were assessed by immunoblotting in MCF-7 cells treated with N64-sh-SFCM or N64C-SFCM. Figure 3D shows that while the levels of the inactive forms of these proteins did not change, the levels of the phosphorylated forms increased in MCF-7 cells treated with N64-sh-SFCM, as compared with their respective control. These factors are known to induce the motile and invasive capacities of cancer cells through the promotion of the epithelial–mesenchymal transition (EMT). To further confirm this transition, we assessed the expression levels of epithelial and mesenchymal markers in MCF-7 cells exposed to SFCM from N64-sh and N64C cells. N64-sh-SFCM decreased the level of EpCAM and E-cadherin, and up-regulated N-cadherin, vimentin, ZEB1 and Twist1 (important mesenchymal markers), as compared to control cells (Figure 3D). Together, these results indicate that ATR down-regulation in breast stromal fibroblasts triggers the EMT process in breast cancer cells in a paracrine manner, which demonstrates their active status.

Next, we sought to investigate the possible role of SDF-1 and IL-6 in these paracrine pro-invasive/migratory effects of ATR-deficient fibroblasts. To do this, N64-sh-SFCM was challenged either with anti-IgG antibody (used as negative control) or with anti-SDF-1 neutralizing antibody, and the effect on the migration/invasion abilities of the MCF-7 cells were studied as described above. Figure 3E confirms the pro-migratory/invasive effects of N64sh-SFCM relative to N64C-SFCM. However, the neutralizing anti-SDF-1 antibody repressed these paracrine effects (Figure 3E). We have subsequently inhibited IL-6 in N64-sh-SFCM using 2 different concentrations of neutralizing anti-IL-6 antibody (30 and 300 ng/ml) for 24 h, IgG was utilized as control. Figure 3F shows that the inhibition of IL-6 significantly reduced the migration/invasion abilities of MCF-7 cells in a concentration-dependent manner. On the other hand, addition of recombinant IL-6 to SFM enhanced the migration/invasion abilities of MCF-7 cells in a dose-dependent manner (Figure 3F). These results indicate that SDF-1 and IL-6 are potent mediators of the paracrine pro-migratory/invasiveness capacities of ATR-deficient breast stromal fibroblasts.

### ATR-deficient fibroblasts stimulate breast cancer orthotopic tumor growth in mice

**Figure 4:**
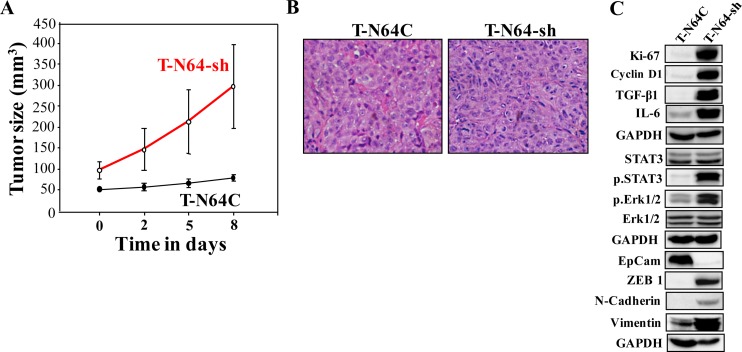
ATR-deficient breast fibroblasts enhance breast orthotopic tumor xenograft formation and growth Breast cancer orthotopic xenografts were created by co-injecting MDA-MB-231 cells with N64-sh cells (T-N64-sh) or N64C cells (T-N64C) under the nipples of nude mice (n=5). **(A)** Graph showing tumor sizes. Error bars represent means ± S.D. **(B)** Tissues were subjected to hematoxylin and eosin staining (Envision 40x). **(C)** Tumors were excised and whole-cell lysates were prepared and protein levels were assessed by immunoblotting using the indicated antibodies.

To investigate the effect of ATR deficiency in human breast fibroblasts on tumor growth *in vivo*, 10 nude mice were randomized into two groups (n=5) and orthotopic breast tumor xenografts were created under the nipples by co-implantation of MDA-MB-231 cells (2×10^6^) with either N64-sh or N64C cells (2×10^6^). All mice developed tumors. However, tumors bearing ATR-deficient fibroblasts (T-N64-sh) appeared earlier and grew vigorously faster relative to those having control cells (T-N64C) (Figure 4A). This indicates that ATR-deficient BSFs can enhance orthotopic tumor growth in mice.

We have next excised tumors and performed hematoxylin and eosin staining for histopathological analysis. Figure 4B shows abundant nuclei spindle shape in T-N64-sh as compared to T-N64C. In addition, T-N64-sh tissues showed no tubular structures, which were clearly developed in T-N64C tissues (Figure 4B). This shows that ATR-deficient BSFs enhance tumor aggressiveness.

In addition, Figure 4C shows that T-N64-sh tumors express higher level of the proliferation marker Ki-67, as well as cyclin D1 than the control tissue. Likewise, the levels of IL-6 and TGF-β1 were higher in T-N64-sh than in T-N64C tissues (Figure 4C). Furthermore, N64-sh cells activated STAT3 and Erk1/2 in tumor xenografts (Figure 4C). Figure 4C shows also that while the levels of the mesenchymal markers N-cadherin, ZEB1, and vimentin were higher in the T-N64-sh tumors, EpCAM was down-regulated in these tumors as compared to T-N64C. This shows that the presence of ATR-deficient breast stromal fibroblasts enhanced the EMT process in breast cancer cells in orthotopic tumor xenografts.

### Ectopic expression of ATR inactivates breast myofibroblasts

**Figure 5:**
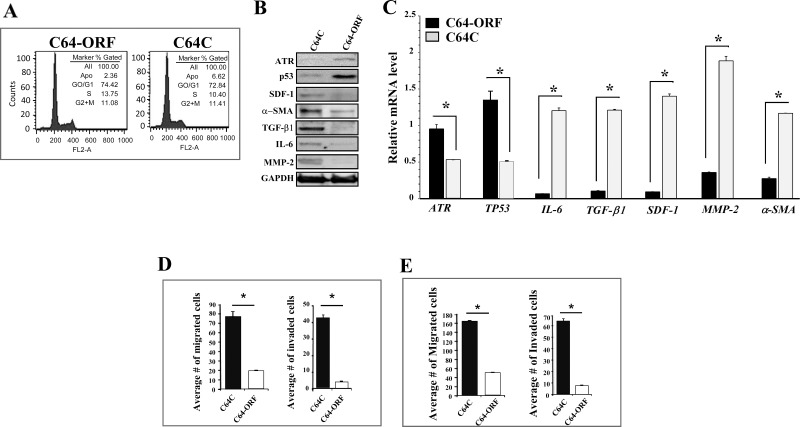
Ectopic expression of ATR suppresses breast myofibroblasts and their paracrine procarcinogenic effects CAF-64 cells were transfected with *ATR*-ORF (C64-ORF) or a control plasmid (C64C). **(A)** cell cycle analysis was performed by flow cytometry. **(B)** Whole-cell lysates were prepared from the indicated cells and were used for immunoblotting analysis. **(C)** Total RNA was extracted from the indicated cells, and the mRNA levels of the indicated genes were assessed by qRT-PCR. Error bars represent means ± SD. ^*^P < 0.01. **(D)** C64-ORF and C64C cells (10^5^) were seeded in SFM onto the upper compartment of the migration and invasion plates and incubated for 24 h in the presence of CpM in the lower chamber of 24-well BD BioCoat plates. The number of migrated/invaded cells was represented in histograms. Error bars represent mean ± SD (n=3). ^*^P < 0.0003. **(E)** MCF-7 cells were seeded with SFCM from the indicated cells onto the upper compartment of the migration and invasion plates, and then were incubated for 24 h. Error bars represent mean ± SD. ^*^P< 1.3x10^−6^.

To further confirm the role of ATR in suppressing the procarcinogenic characteristics of breast stromal fibroblasts, CAF-64 cells were transfected either with a plasmid expressing *ATR*-ORF (C64-ORF) or the corresponding empty vector (C64C). Figure 5A shows similar number of cells in the various phases of the cell cycle in both cell cultures, indicating that ATR up-regulation did not affect the cell cycle distribution. The immunoblotting analysis confirmed the increase in the ATR protein level in C64-ORF cells as compared to C64C cells (Figure 5B). This led to potent p53 up-regulation, and the down-regulation of the SDF-1, α-SMA, TGF-β1, IL-6 and MMP2 proteins in C64-ORF as compared to the control cells (Figure 5B). Similar results were obtained at the mRNA level (Figure 5C). Moreover, ectopic expression of ATR markedly reduced both the invasion and the migration abilities of breast stromal fibroblasts as compared with control cells (Figure 5D). We have next tested the paracrine effects of these cells on the invasion/migration abilities of breast cancer cells. To this end, MCF-7 cells were incubated in the presence of SFCM from C64-ORF or C64C, and then their migration/invasion abilities were tested as described above. Figure 5E shows that the expression of ATR in stromal fibroblasts strongly suppressed the migration/invasion abilities of breast cancer cells in a paracrine manner. Together, these results confirm that ATR expression in breast stromal fibroblasts inhibits these cells and suppresses their paracrine pro-carcinogenic effects.

### The effects of ATR-deficiency are p53-dependent

**Figure 6:**
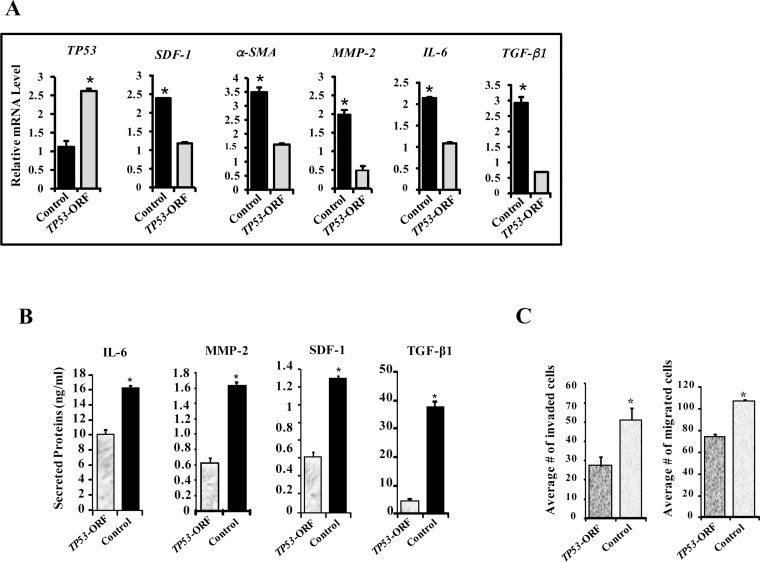
ATR down-regulation activates breast stromal fibroblasts in a p53-dependent manner N64-sh cells were transfected with a vector bearing *TP53*-ORF or an empty vector used as control. **(A)** Total RNA was extracted from the indicated cells, and the mRNA levels of the indicated genes were assessed by qRT-PCR. Error bars represent mean ± S.D (n=3). ^*^P< 0.017. **(B)** SFCM from the indicated cells were collected, and then the secreted levels of the indicated proteins were determined by ELISA. Error bars represent mean ± S.D (n=3). ^*^P< 0.0001. **(C)** The migration and invasion abilities of the indicated cells were assessed as described in Figure 3F. Error bars represent mean ± S.D (n=3). ^*^P< 0.009.

p53 is a downstream effector of ATR, and we have shown here that p53 expression is modulated in an ATR-dependent manner (Figures 2A and 5B, 5C). Furthermore, p53 plays major roles in repressing breast stromal fibroblasts through regulating the expression of several important genes such as SDF-1 and IL-6 [13, 14]. Therefore, we decided to investigate the role of p53 in the ATR-deficiency related effects. Therefore, ATR-deficient cells (N64-sh) were transfected with adenovirus-based plasmids expressing *TP53*-ORF (open reading frame) or a control plasmid. Figure 6A shows clear increase in the *TP53* mRNA (2.5 fold) in cells expressing *TP53*-ORF as compared to the controls. On the other hand, mRNA levels of α-SMA, SDF-1, MMP-2, IL-6 and TGF-β1 were significantly reduced in N64-sh cells expressing *TP53*-ORF as compared to their respective controls (Figure 6A). Similarly, the secreted levels of IL-6, MMP-2, SDF-1 and TGF-β1 were also significantly reduced in N64-sh cells expressing *TP53*-ORF (Figure 6B). This indicates that ectopic expression of p53 inhibited the effects of ATR-deficiency on the expression/secretion of myofibroblast-related genes.

Furthermore, *TP53* up-regulation significantly reduced the invasiveness and the migratory capabilities of N64-sh cells as compared to their corresponding control cells (Figure 6C). These results indicate that p53 is involved in the ATR-dependent repression of breast stromal fibroblasts.

## DISCUSSION

We have shown here that ATR plays a major role in suppressing BSFs and their pro-carcinogenic effects both *in vitro* and *in vivo*. Indeed, specific down-regulation of ATR increased the proliferation and the migration/invasion abilities of BSFs and also enhanced the expression of the 2 major myofibroblast markers α-SMA and SDF-1. In addition, TGF-β1, IL-6 and MMP-2 were also up-regulated in ATR-deficient BSFs. These ATR-related effects were inhibited upon ectopic expression of ATR or p53, which indicates that p53 mediates the ATR effects in BSFs. p53 is a well known tumor suppressor gene, which also inhibits BSF through repressing the expression of SDF-1 and IL-6 [13, 14]. Therefore, p53 has also non-cell-autonomous tumor suppressor function [15, 16], and thereby, ATR may also play such role.

The active status of ATR-deficient BSFs was next confirmed by showing that specific down-regulation of ATR enhances the EMT process in breast cancer cells in a paracrine manner. The non-cell-autonomous ATR-related enhancement of the migration/invasion abilities of breast cancer cells was mediated in an SDF-1/IL-6-dependent manner. These 2 cytokines play major roles in the pro-carcinogenic effects of active BSFs [3, 4]. The pro-carcinogenic effects of ATR-deficient breast stromal fibroblasts were corroborated both *in vitro* using direct coculturing in engineered human breast tissues composed of breast cancer cells and BSFs, and also *in vivo* through the formation of orthotopic tumors by co-implantation of breast cancer cells and BSFs. In both cases ATR-deficient cells enhanced the growth of breast cancer cells, which was confirmed by showing the expression of high levels of Ki-67 and cyclin D1. *In vivo*, the presence of ATR-deficient BSFs generated more aggressive tumors wherein STAT3 was highly active and the pro-metastatic genes TGF-β1 and IL-6 were highly expressed, and the EMT process was induced. Together, these results indicate that ATR down-regulation in BSFs has potent non-cell-autonomous procarcinogenic effects. Similar effects were previously shown in other tumor suppressor proteins such as p16, PTEN, pRB and CAV-1 [5, 6]. This indicates that several genes repress the devastating procarcinogenic effects of breast stromal fibroblasts, and that the down-regulation of one of these genes is sufficient to trigger the pro-tumorigenic process through paracrine signaling.

Importantly, we have also shown that ATR is down-regulated in 9 out of 12 (75 %) CAFs as compared to their corresponding adjacent TCFs isolated from the same breast at adjacent histologically normal tissues. This differential expression suggests that ATR down-regulation in CAFs is due to the presence of these cells close to cancer cells, which perturbs the microenvironment through paracrine signaling. Indeed, we have recently shown that cancer cells activate breast stromal fibroblasts in an IL-6-dependent manner [3]. This confirms that ATR is another important target of breast cancer cells in BSFs, and that this protein kinase plays key roles in the carcinoma-stroma cross-talk during breast carcinogenesis.

## MATERIALS AND METHODS

### Cells and cell culture

Breast fibroblast cells were obtained and used as previously described [17]. MCF-7, MDA-MB-231 and MCF-10A cells were purchased from ATCC and were authenticated using short tandem repeat profiling by ATCC, propagated, expanded, and frozen immediately into numerous aliquots after arrival. The revived cells were utilized within 10 to 12 passages and not exceeding a period of 3 months, and were cultured following the instructions of the company. Cells were regularly screened for mycoplasma contamination using MycoAlert Mycoplasma Detection Kits (Lonza). All supplements were obtained from Sigma (Saint Louis, MO, USA) except for antibiotic and antimycotic solutions, which were obtained from Gibco (Grand Island, NY, USA). Cells were maintained at 37°C in humidified incubator with 5% CO_2_. Human IL-6 recombinant protein (hBA-184) (Santa Cruz, CA). The anti-IL-6 monoclonal antibody (6708.11) was purchased from Sigma-Aldrich, USA. Anti-SDF-1 neutralizing antibody (Human CXCL12/SDF-1 antibody) and IgG (6-101-C-ABS) from R&D Systems.

### Cellular lysate preparation and immunoblotting

This has been performed as previously described [18]. Antibodies directed against alpha smooth muscle actin (α-SMA), Ki-67, transforming growth factor beta 1 (TGF-β1), Stromal-derived factor-1 (SDF-1), Twist-1, Vimentin (RV202), MMP-9, (ab38898), ATR (ab54793), N-cadherin and interleukin-6 (IL-6) were purchased from Abcam (Cambridge, MA); STAT3, pSTAT3-Tyr705 (D3A7), Snail (C15D3), E-cadherin (24E10), EpCAM (UV1D9), JAK-2 (D2E12) and phospho-JAK-2 (TYR1007/1008), CyclinD1 (2922), Akt, phospho-Akt (193H12), MMP-2 (4022), ERK1/2 (137F5) and phospho-ERK1/2 from Cell Signaling (Danvers, MA); ZEB-1 (4C4) from Abnova (Taipei, Taiwan); p21 (F-5), p53 (DO-1) and Glyceraldehydes-3-phosphate dehydrogenase (GAPDH, FL-335) were purchased from Santa Cruz (Santa Cruz, CA).

### RNA purification, RT-PCR and qRT-PCR

Total RNA was purified using the TRI reagent (Sigma) according to the manufacturer's instructions, and was treated with RNase-free DNase before cDNA synthesis using the RT-PCR Kit (Clontech, USA). cDNA was amplified using the Platinum® *Taq* DNA Polymerase (Invitrogen). The RT^2^ Real-Time™ SYBR Green qPCR mastermix (Roche, Germany) was used and the amplifications were performed utilizing the light cycler 480 (Roche, Germany). The melting-curve data were collected to check PCR specificity, and the amount of PCR products was measured by threshold cycle (Ct) values and the relative ratio of specific genes to *GAPDH* for each sample was then calculated. The respective primers are:

*TP53*: 5′-CAGTCTACCTCCCGCCATAA-3′ and 5′- CCACAACAAAACACCAGTGC-3′

*MMP-2*: 5′-CATGTCGCCCCTAAAACAGA-3′ and 5′-CCATCAAACGGGTATCCATC-3′

*GAPDH*: 5′-GAGTCCACTGGCGTCTTC-3′ and 5′-GGGGTGCTAAGCAGTTGGT-3′

*SDF-1*: 5′-TAGTCAAGTGCGTCCACGA-3′ and 5′-GGACACACCACAGCACAAAC-3′

*α-SMA*: 5′-CCGACCGAATGCAGAAGGA-3′ and 5′-ACAGAGTATTTGCGCTCCGAA-3′

*TGF-β1*: 5′-TGTGTGCTGAAGCCATCGTTG-3′ and 5′-CCGGCTTGTCTGAAAAGGTCA-3′

*IL-6*: 5′-GACAAAGCCAGAGTCCTTCAGAGA-3′ and 5′-CTAGGTTTGCCGAGTAGATCT-3′

*ATR*: 5′-GTCATATACACTCCCTTTTCTTTA-3′ and 5′-GTCATATACACTCCCTTTTCTTTA-3′

### ATR-shRNA transfection

*ATR*-shRNA (KHD1318) expressed in sure silencing shRNA plasmid and the corresponding control plasmid were obtained from GenScript Corporation, and were used to carry out transfection using human dermal fibroblast nucleofector 2000 transfection kit (Invitrogen) following the protocol recommended by the manufacturer.

### Viral infection

Lentivirus based vectors bearing *TP53*–ORF as well as the respective control (Origene) were used to prepare the corresponding lentiviral supernatants from 293FT cells. Lentiviral supernatants were collected 48 h post-transfection, filtered and used for infection. Cells were infected for 48 h, and then media were replaced with complete media and cells were grown for 3 days.

### *ATR*-ORF

Lentivirus based vectors bearing *ATR*-ORF, (pLenti-C-Myc-DDK) and the corresponding control were used to carry out transfection using human dermal fibroblast nucleofector 2000 transfection kit (Invitrogen) following the manufacturer's recommendations. After ten days of transfection, cells were tagged using OriGene anti-DDK antibody (TA50011) and sorted with BD FACSAria cell sorting system.

### ELISA assays

Supernatants from 24 h fibroblast cell cultures were harvested and ELISA was performed according to the manufacturer's instructions (R&D Systems). The OD was used at 450-nm on a standard ELISA plate-reader. These experiments were performed in triplicates.

### Chemotaxis and invasion assays

The 24-well BD BioCoat Matrigel Invasion chambers were used as per the manufacturer guideline (BD Bioscience). 2-4 × 10^5^ cells were added to the upper wells separated by an 8 micron pore size PET membrane with a thin layer of matrigel basement membrane matrix (for invasion) or without (for migration). The membranes were stained with Diff Quick stain (Fisher Scientific) after removing the non-migrated cells from the top of the membrane with Q-tips. After air-drying, the membranes were cut and mounted on slides with oil, and cells that had migrated to the underside of the filter were counted using light microscope (Zeiss Axio Observer) in five randomly selected fields (magnification; 40x). Each assay was performed in triplicate.

### Cell proliferation

This assay was performed in a real-time and label-free manner using the xCELLigence RTCA technology (Roche, Germany) that measures impedance changes in a meshwork of interdigitated gold microelectrodes located at the bottom well (E-plate). Exponentially growing cells (2×10^4^) were seeded in E-plate with complete medium as per manufacturer's instruction. All data were recorded and analyzed by the RTCA software. Cell Index (CI) was used to measure the change in the electrical impedance divided by the background value to represent cell status. Each assay was performed in triplicate.

### Conditioned media

Cells were cultured in medium without serum for 24 h, and then media were collected and briefly centrifuged. The resulting supernatants were used either immediately or were frozen at −80°C until needed.

### Immunohistochemistry staining on FFPE tissues

Immunohistochemistry for ATR was done on formalin-fixed, paraffin-embedded tissues using anti-ATR antibody from abcam (ab54793) at a dilution of 1:500 and tissues were stained using automated staining platform (Ventana). Envision + polymer (ready to use; Dako) was used as a secondary antibody. Color was developed with 3,3′-diaminobenzidine (DAB) and instant hematoxylin (Shandon) was used for counterstaining.

### Orthotopic tumor xenografts

Animal experiments were approved by the KFSH&RC institutional Animal Care and Use Committee (ACUC) and were conducted according to relevant national and international guidelines. 10 female nude mice were randomized into 2 groups and breast cancer orthotopic xenografts were created by coimplantation of MDA-MB-231 cells (2×10^6^) with N64-sh, or N64C cells (2×10^6^) under the nipple of each mouse. Tumor size was measured with a caliper using the following formula (Length X Width X Height).

### Statistical analysis

Statistical analysis was performed by student's t-test and *P* values of 0.05 and less were considered as statistically significant.
